# Recent Advances in Flexible Materials for Wearable Optical Biosensors

**DOI:** 10.3390/bios15090611

**Published:** 2025-09-16

**Authors:** Linyan Xie, Kai Yang, Mengfei Wang, Wenli Hou, Qiongqiong Ren

**Affiliations:** 1 School of Mathematical Medicine and School of Medical Engineering, Henan Medical University, Xinxiang 453003, China; 13525007916@163.com (K.Y.); 15237138232@163.com (M.W.); 15237315364@163.com (W.H.); 2 Xinxiang Key Laboratory of Neurobiosensor, Xinxiang 453003, China; 3 Henan Engineering Technology Research Center of Neural Sensing and Control, Xinxiang 453003, China

**Keywords:** flexible materials, wearable biosensors, optical sensing, health monitoring, disease prevention

## Abstract

The integration of flexible materials with optical sensing technologies has advanced wearable optical biosensors, offering significant potential in personalized medicine, health monitoring, and disease prevention. This review summarizes the recent advancements in flexible materials for wearable optical biosensors, with a focus on materials such as polymer substrates, nanostructured materials, MXenes, hydrogels, and textile-based integrated platforms. These materials enhance the functionality, sensitivity, and adaptability of sensors, particularly in wearable applications. The review also explores various optical sensing mechanisms, including surface plasmon resonance (SPR), optical fiber sensing, fluorescence sensing, chemiluminescence, and surface-enhanced Raman spectroscopy (SERS), emphasizing their role in improving the detection capabilities for biomarkers, physiological parameters, and environmental pollutants. Despite significant advancements, critical challenges remain in the fabrication and practical deployment of flexible optical biosensors, particularly regarding the long-term stability of materials under dynamic environments, maintaining reliable biocompatibility during prolonged skin contact, and minimizing signal interference caused by motion artifacts and environmental fluctuations. Addressing these issues is vital to ensure robustness and accuracy in real-world applications. Looking forward, future research should emphasize the development of multifunctional and miniaturized devices, the integration of wireless communication and intelligent data analytics, and the improvement of environmental resilience. Such innovations are expected to accelerate the transition of flexible optical biosensors from laboratory research to practical clinical and consumer healthcare applications, paving the way for intelligent health management and early disease diagnostics. Overall, flexible optical biosensors hold great promise in personalized health management, early disease diagnosis, and continuous physiological monitoring, with the potential to revolutionize the healthcare sector.

## 1. Introduction

With the rapid advancement of technology, sensor technologies have become increasingly important in various fields, including healthcare, environmental monitoring, and industrial automation. The core function of sensors is to detect and respond to changes in physical or chemical quantities, converting these measurements into detectable signals. Biosensors, a subset of this field, combine biological recognition elements with physicochemical transducers, enabling them to specifically recognize biomolecules and convert their interactions into detectable signals. This capability provides efficient and accurate detection, making biosensors indispensable in areas such as medical diagnostics, environmental pollution monitoring, and food safety [1,2,3]. They play a critical role in the fields of medicine, life sciences, and public health by enabling precise, reliable, and rapid analysis of contaminants or target analytes [4,5]. Biosensors can be classified according to different criteria. From the perspective of the biological recognition element, they are typically divided into enzymatic sensors, immunosensors, nucleic acid-based sensors, microbial sensors, and whole-cell biosensors. Based on the transduction mechanism, biosensors can be grouped into electrochemical, optical, piezoelectric, and thermal biosensors [6,7].

Optical biosensors, a significant branch of biosensors, utilize optical signals (such as absorption, fluorescence, and surface plasmon resonance) to detect changes in biomolecule interactions. Optical biosensing technology offers several advantages, including high sensitivity, real-time monitoring capabilities, and generally non-invasive detection features. However, it is important to note that not all optical sensors are completely non-invasive. For example, certain surface plasmon resonance (SPR)-based sensors may require direct contact with the skin or even minimally invasive procedures. Additionally, the sensitivity of optical biosensors is highly dependent on the specific sensing mechanism employed, such as SPR, fluorescence, or chemiluminescence, each exhibiting varying degrees of sensitivity depending on the application and design [4]. Nevertheless, compared with other common sensing modalities such as piezoresistive, piezoelectric, and capacitive sensors, optical biosensors provide distinctive benefits. Piezoresistive and capacitive sensors are often susceptible to baseline drift, electrical noise, and limited long-term stability under humid or high-temperature conditions [5,8]. Piezoelectric sensors, although highly sensitive to mechanical stimuli, typically require rigid substrates and are less compatible with flexible and skin-conformal designs [9]. In contrast, optical biosensors enable label-free detection, are inherently immune to electromagnetic interference, and allow real-time monitoring with high specificity even in dynamic environments [1]. Moreover, their capability for multiplexed detection through spectral separation and compatibility with miniaturized optical fibers or waveguides provides a pathway toward wearable, non-invasive, and multi-analyte monitoring. These features collectively highlight why optical biosensing has become a leading modality for the next generation of flexible wearable biosensors. With the continuous advancement of optical materials, sensor design, and integration technologies, the performance of optical biosensors has significantly improved, providing robust support for the expansion of their application areas [10].

In recent years, wearable optical biosensors have emerged as an important development direction within the field of optical biosensors, benefiting from the widespread application of flexible materials [11]. Researchers have designed more flexible, more comfortable, and more efficient sensors by combining flexible materials with optical technologies. Compared with traditional diagnostic tools, wearable optical biosensors have advantages in terms of sensitivity, portability, and user comfort, making them highly suitable for long-term monitoring in daily life and clinical applications [2]. These sensors can continuously monitor physiological parameters, disease biomarkers, and metabolite levels in real time [12], which is particularly important for the early detection of chronic diseases such as diabetes and cardiovascular diseases. In these diseases, timely and accurate monitoring of biomarkers (e.g., glucose, lactic acid, blood oxygen saturation) can significantly improve patient prognosis and provide innovative solutions for health management, disease early warning, and personalized medicine [1,13,14]. Therefore, researching flexible materials that can enhance sensor stability, biocompatibility, and signal transmission performance is of great significance to both the academic and industrial communities.

In parallel with academic research, leading companies such as Rockley Photonics and Siphox Health are pushing forward the commercialization of wearable optical biosensors, especially for applications in continuous health monitoring, fitness tracking, and preventive healthcare. Their active involvement highlights the growing industrial and societal demand for reliable wearable biosensing technologies, providing strong evidence of the field’s increasing importance.

However, despite the significant application potential of flexible optical biosensors in various fields, several challenges remain. First, the long-term stability of sensors and their reliability in dynamic environments still require further improvement, especially in terms of how flexible materials perform under continuous motion, temperature fluctuations, and external pressure [15]. Second, the sensitivity and selectivity of sensors, particularly in the detection of low-concentration analytes, still face certain technological bottlenecks [9]. Lastly, balancing sensor performance with comfort and resolving the adaptation issues between the materials and human skin are important areas of ongoing research [16].

This review aims to provide an overview of the latest technological advancements in flexible optical biosensors, with a particular focus on the application and innovation of flexible materials in sensor design. By summarizing the current advantages and limitations of existing technologies, this paper will offer insights into future research directions, aiming to provide valuable references for academic research and industrial applications in related fields. Furthermore, this review seeks to promote the widespread use of flexible optical biosensors in medical, health monitoring, and disease prevention applications.

The following chapter will delve into technological innovations in flexible materials, systematically introducing polymer substrates, nanostructured materials, MXenes, hydrogels, and textile-based platforms, and how their unique properties (e.g., stretchability, biocompatibility, optical tunability) lay the groundwork for advanced wearable optical sensing technologies.

## 2. Technological Innovations in Flexible Materials

**Figure 1:**
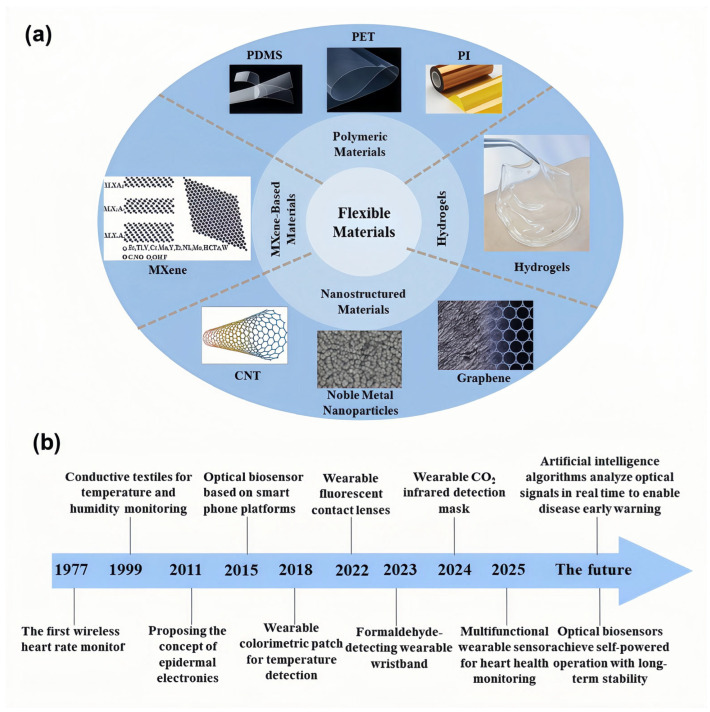
Overview of Flexible Materials for Wearable Sensors. (**a**) Schematic overview of major categories of flexible materials used in wearable optical biosensors, including polymeric materials (e.g., PDMS, PET, PI), hydrogels, nanostructured materials (e.g., CNT, noble metal nanoparticles, graphene), and MXene-based materials. These materials offer excellent biocompatibility, mechanical flexibility, and functional tunability, enabling their wide application in next-generation wearable sensing platforms. (**b**) Timeline illustrating the evolution of wearable sensor technologies. From the first wireless heart rate monitor to the integration of artificial intelligence and self-powered systems, this timeline highlights key milestones and the growing sophistication of biosensor capabilities for health monitoring and personalized care.

Flexible materials have become a crucial component of modern sensor systems due to their excellent biocompatibility, mechanical adaptability, and lightweight nature. These materials enhance the performance and comfort of sensors, driving advancements in non-invasive detection, real-time monitoring, and personalized healthcare technologies [17,18,19]. To meet the demands of multi-signal recognition in complex environments, sensor materials are evolving toward transparency, stretchability, bendability, and even foldability [20]. This trend has accelerated the widespread application of flexible sensors. Currently, these sensors are typically composed of high-performance polymer materials [7], nanomaterials [21], and their composite structures [22], which exhibit tremendous potential in flexible electronic devices (Figure 1a). To further illustrate the significance of these flexible materials in sensor applications, Figure 1b presents a timeline that tracks the development and evolution of wearable sensor technologies. The timeline highlights significant milestones, from the introduction of wireless heart rate monitors to the incorporation of advanced materials such as graphene and the integration of AI-driven systems. This progression underscores the increasing sophistication and potential of flexible sensors in personalized healthcare and real-time monitoring [23,24].

### 2.1. Polymer Substrates and Their Derivatives

Polymeric materials, also known as polymers, are widely used in flexible substrates, electrodes, and semiconductor layers due to their excellent design flexibility and outstanding physical properties [25,26,27]. Common flexible substrate materials include polydimethylsiloxane (PDMS), polyimide (PI), and polyethylene terephthalate (PET). Among these, PDMS is favored in many sensor applications due to its exceptional flexibility, optical transparency, and good biocompatibility. Its low surface energy helps maintain the cleanliness of the sensor surface, although its low electrical conductivity necessitates composite incorporation with conductive materials to enhance performance [28]. Polyimide (PI) is commonly chosen for functional substrates because of its excellent thermal stability, insulation properties, and film-forming ability [29]. PET, a thermoplastic polyester, offers good mechanical strength and thermal stability, making it widely used in wearable fabrics and surface-attached sensors [30].

**Figure 2:**
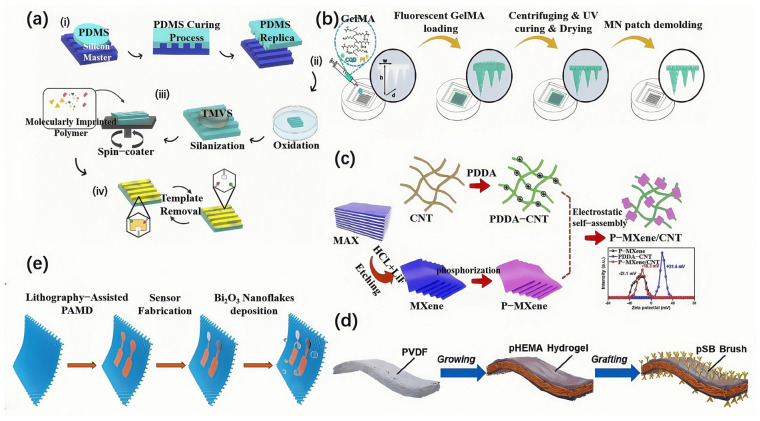
Representative applications of various flexible materials in wearable optical biosensors. (**a**) Fabrication process of molecularly imprinted photonic sensors using a patterned PDMS substrate for selective detection of chlorpyrifos (CPR), demonstrating template-guided recognition capability [31]. (**b**) Schematic illustration of the preparation of fluorescent microneedle (MN) patches using graphene-based materials and GelMA. The patches enable real-time, high-sensitivity detection of bilirubin, suitable for non-invasive monitoring of neonatal jaundice [33]. (**c**) Construction of a phosphorus-doped MXene (P-MXene)/carbon nanotube (CNT) composite sensor via electrostatic self-assembly. The resulting hybrid sensor exhibits enhanced conductivity, lower detection limits, and improved sensitivity [34]. (**d**) A flexible epidermal secretion-purified biosensing patch uses a PVDF microfiltration membrane as the substrate, and a sebum-resistant membrane is prepared by sequentially modifying a pHEMA hydrogel layer and pSB nanobrush, which can effectively eliminate the interference of sebum and sebum-soluble substances [35]. (**e**) The β-Bi_2_O_3_ is modified on the ion-selective layer via electrochemical methods on the nanoflakes. The resulting nanoflakes possess fast ion conduction function and moisture resistance and can retain their functionality after washing [36].

In recent years, researchers have achieved precise control over the optical and electrical properties of polymer materials by introducing special functional groups, adjusting molecular chain structures, or creating topological structures. For example, block copolymers combine bio-recognition segments with optically active segments, effectively improving selectivity toward biomolecules and enhancing the response of optical signals. Sara Resende and colleagues replicated nano-grating structures within PDMS, creating a photonic detection platform that provides an efficient optical biosensor for C-reactive protein (CRP) detection (Figure 2a) [31]. Li et al. developed a transparent fluorescent dental protector based on zinc oxide-polydimethylsiloxane (ZnO-PDMS) nanocomposites for high-sensitivity detection of volatile sulfur compounds (VSC) in the mouth and precise localization of dental lesions such as cavities and periodontitis. The dental protector uses PDMS as the flexible substrate and combines the fluorescent properties of ZnO quantum dots to selectively respond to VSCs. Experimental results show that the protector accurately identifies lesion locations during 7 h of continuous monitoring and visualizes the lesions through 3D imaging analysis. ZnO-PDMS material demonstrates excellent biocompatibility, with a cell viability rate exceeding 95% after 72 h of cell culture. Moreover, the material has low production costs and significant potential for large-scale applications [32].

### 2.2. Nanostructured Materials

Nanomaterials are materials whose structural units range in size from 1 nm to 100 nm. Due to their unique size effects and surface characteristics, nanomaterials play a critical role in enhancing the sensitivity and response speed of sensors. Depending on their dimensional properties, nanomaterials are typically classified into zero-dimensional (0D, such as nanoparticles), one-dimensional (1D, such as nanotubes and nanowires), and two-dimensional (2D, such as nanosheets and nanoribbons) structures [37]. The dimensional characteristics significantly affect how nanomaterials interact with biological and optical signals, thus offering distinct performance advantages across various sensor applications [38].

Noble metal nanoparticles, such as Ag and Au nanoparticles, have been widely applied in cancer biomarker detection, particularly when combined with surface plasmon resonance (SPR) technology [39]. Compared to traditional diagnostic methods, noble metal nanoparticles enhance optical signals, providing higher selectivity and lower cytotoxicity. These nanoparticles can significantly improve the sensitivity of sensors, thereby enhancing the accuracy and reliability of biomolecule detection [40]. Carbon-based nanomaterials, particularly carbon nanotubes (CNTs) and graphene, have become key materials in modern sensor technologies due to their large specific surface area, excellent electrical properties, and significant mechanical strength. The unique 1D structure of CNTs enables high-sensitivity electronic sensing, and their chemical reactivity can be modulated through surface functionalization, further enhancing sensor performance [41,42,43]. Jia et al. integrated multi-walled carbon nanotubes (MWCNTs) with polyimide aerogel-based paper (CPs) to create a conductive material. By introducing MWCNTs into the multilayered folded structure of CPs, the conductivity and thermal stability were enhanced, ensuring good stability and high-temperature responsiveness after 1000 cycles. This demonstrates great potential for high-temperature-resistant flexible sensors [44].

Graphene, as a typical 2D material, is known for its exceptional electronic mobility, high strength, and transparency, making it an ideal material for optoelectronic sensors. Its large surface area and excellent conductivity have led to its widespread use in biosensors, particularly in optical sensor applications [45,46]. Tabatabaee et al. developed a graphene-based invisible nano-tattoo sensor for non-invasive, real-time monitoring of neonatal jaundice (Figure 2b). The sensor delivered fluorescent carbon quantum dots to the dermal layer, enhancing bilirubin detection sensitivity by combining the superior optical cross-linking properties and biocompatibility of graphene. In a mouse skin model, the sensor successfully achieved 6 h of dynamic monitoring with a detection limit of 0.5 mg/dL, and the results were highly correlated with blood bilirubin concentrations. These nanomaterials, especially those with unique dimensional structures, have significantly advanced the field of biosensors, providing high-sensitivity, non-invasive, and real-time monitoring solutions for biomedical applications [33].

### 2.3. MXene-Based Materials

MXenes are a novel class of 2D transition metal carbides and nitrides (M_n+1_X_n_T_x_), derived from MAX-phase materials through selective etching. These MXene materials possess a layered structure, hydrophilic surface termini, high conductivity, and excellent mechanical and chemical stability, making them highly suitable for sensor applications [47,48]. The surface plasmon resonance enhancement, optical signal amplification, and biomolecular recognition functionalities of MXenes are primarily attributed to their plasmonic behavior in the visible to near-infrared spectrum [49]. To improve their sensor performance, the surface properties of MXenes can be modified, which in turn alters their interlayer spacing and chemical interactions [50].

For example, Zr-TCBPE-MOL/MXene composite materials have been employed in enhanced electrochemiluminescence (ECL) biosensors, successfully applied in the detection of microRNA-141 [51]. Additionally, Yang et al. developed phosphorus-doped MXene (P-MXene) crosslinked with carbon nanotubes (CNTs) through chemical vapor deposition (CVD) and electrostatic assembly. The crosslinking of P-MXene with CNTs effectively alleviates stacking effects and accelerates ion transport, enhancing the electrochemical performance of the material (Figure 2c). The P-MXene/CNT-based planar micro-supercapacitors exhibit excellent mechanical flexibility and integration capabilities, with the fabricated micro-sensors demonstrating low detection limits and high sensitivity [35]. Moreover, MXene materials can also function as fluorescence quenchers, owing to their broad-spectrum absorption characteristics, enabling the implementation of an internal filter effect (IFE)-based sensing strategy. For instance, the fluorescence of Cu nanoclusters is quenched and subsequently restored upon interaction with MXene interfaces and glutathione. This unique property makes MXenes highly promising for applications in optical sensors, expanding their potential in biosensing and other advanced optical detection technologies [52].

### 2.4. Hydrogels and Conductive Composites

Hydrogels are polymer networks with three-dimensional crosslinking, capable of absorbing large amounts of water while maintaining structural stability [53,54]. This property makes them widely applicable in fields such as biomedicine, environmental monitoring, and wearable sensors. Both natural polymers (such as gelatin and sodium alginate) and synthetic polymers (such as PEG and PAM) can be engineered to respond to external factors such as pH, temperature, and ionic strength, allowing for tailored functionality [55,56].

Conductive hydrogels, especially those with self-healing, stretchable, and transparent properties, are particularly suited for wearable strain sensors. For example, Qing et al. developed a PVA/pullulan/borax hydrogel, which demonstrates rapid self-healing, high transparency (92.9%), and multi-sensing capabilities [57]. Zhang et al. synthesized PAHS hydrogel via UV-induced free radical polymerization, incorporating Al_2_O_3_ nanoparticles and LiCl to achieve conductivity, transparency (450–800 nm transmission > 80%), high stretchability (up to 800%), and antifreeze properties [58]. Wang et al. developed a flexible epidermal secretion-purified biosensing patch. The sebum-resistant membrane is prepared by sequentially modifying a poly(hydroxyethyl methacrylate) (pHEMA) hydrogel layer and poly (sulfobetaine) (pSB) nano-brush on a polyvinylidene fluoride (PVDF) microfiltration membrane as the substrate. It can effectively eliminate the interference of sebum and sebum-soluble substances. The pHEMA enhances hydrophilicity and blocks sebum penetration, while the pSB nano-brush further eliminates interference through self-cleaning capability (standing upright when in contact with liquid and pushing sebum away). This patch improves the monitoring accuracy of uric acid, pH value, and sodium ions in sweat by up to 12% (Figure 2d) [35]. These hydrogels not only offer excellent adhesion and fatigue resistance but also enable stable monitoring of various physiological signals under different environmental conditions. This demonstrates their enormous potential for high-performance biosensors.

### 2.5. Textile-Based Platforms and Hybrid Composites

Advances in wearable sensors have been driven by the application of textile-compatible materials. Zhang et al. developed a Ti_3_C_2_T_X_-MXene/PET textile that features a DNA-like helical structure and a three-dimensional conductive network, allowing it to respond rapidly to mechanical deformations such as stretching, bending, and compression [59]. Shi et al. developed a washable textile biosensor based on β-Bi_2_O_3_ nanoflakes for epidermal sweat detection. Due to their unique crystal structure, these nanoflakes possess fast ion conductivity and moisture resistance, enabling the sensor to maintain high sensitivity (58.70 mV/dec) while retaining over 90% of its performance after 20 washing cycles and more than 70% after 50 washing cycles. By integrating a wireless transmission module, a wearable wristband was fabricated, realizing real-time monitoring of sodium ions in sweat during exercise, which solves the problem that traditional textile sensors are difficult to balance washability and sensitivity (Figure 2e) [36]. Furthermore, Zhang et al. developed a flexible smart textile sensor based on side-emitting polymer optical fibers (POF), capable of continuously monitoring pulse and arterial oxygen saturation (SpO_2_). The sensor significantly improves emission efficiency through the micro-bending protrusion structure of the POF and remains stable under dynamic motion and sweating conditions, making it suitable for long-term wear [60]. Li et al. developed a “fabric-SCF-silicone” sandwich-structured sensor that combines the micro-bending sensitivity of the silicone film with the breathability of the fabric substrate, enabling seamless monitoring of fingertip pulse and neck muscle movement. The sensor’s low-pressure sensitivity reaches 2.2 kPa^−1^, significantly outperforming traditional PDMS-based optical sensors. It can precisely capture heart rate variability and muscle fatigue signals during athletes’ training [61]. These textile-integrated platforms highlight the importance of combining mechanical design with advanced nanomaterials, providing strong support for scalable wearable sensor solutions.

### 2.6. Thin-Film of Inorganic Non-Metallic Material

In addition, thin films of inorganic non-metallic materials such as semiconductors and dielectrics have recently emerged as an important class of flexible materials for wearable optical biosensors. Although these materials are conventionally brittle, when engineered in ultra-thin film form, they exhibit remarkable flexibility, making them suitable for conformal biosensing applications. Recent studies have demonstrated that flexible thin-film semiconductors can be integrated into wearable devices to provide stable optoelectronic performance under repeated bending and stretching. For example, Li et al. proposed a monolithic integration method for manufacturing flexible photonic devices based on chalcogenide glass (ChG). They successfully fabricated devices such as waveguides, micro-disk resonators, vertically coupled add-drop filters, and 3D woodpile photonic crystals, and these devices possess both excellent optical performance and extreme mechanical flexibility. This method adopts low-cost ultraviolet contact lithography and is compatible with multiple ChG compositions (e.g., Ge_23_Sb_7_S_70_, As_2_Se_3_, As_2_S_3_), laying a foundation for 3D flexible photonic integration and the application of flexible biosensors [62]. Notaros et al. proposed and experimentally validated the first 300-mm wafer-scale fabrication platform and process for mechanically flexible integrated photonics. This platform uses silicon nitride (Si_3_N_4_) and silicon dioxide (SiO_2_) as its optical functional materials, with polyester film serving as the flexible supporting substrate. Being compatible with CMOS technology, the platform enables the fabrication of flexible photonic wafers and chips. It realizes key optical functionalities at visible wavelengths, including chip coupling, waveguide routing, and passive devices, thereby laying a foundation for extending integrated photonics into new application areas such as wearable healthcare monitoring and flexible displays [63]. These studies highlight the potential of inorganic thin films to expand the material toolbox for biosensor fabrication, combining the excellent optical and electronic properties of inorganic materials with the flexibility required for wearable applications.

In summary, advances in flexible materials have significantly broadened the functional landscape of wearable optical biosensors. Polymer substrates, nanostructured materials, MXene composites, hydrogels, and textile-based platforms offer a robust foundation for next-generation sensor design. These materials provide complementary properties-such as flexibility, biocompatibility, conductivity, and integration potential-supporting stable performance under complex physiological conditions. Table 1 provides a comparative overview of these representative materials, highlighting their key features and limitations to guide informed material selection and device optimization.

The next chapter will focus on optical sensing mechanisms for wearable biosensors, detailing how surface plasmon resonance (SPR), optical fiber sensing, fluorescence, chemiluminescence, and surface-enhanced Raman spectroscopy (SERS) work in synergy with flexible materials to achieve high-precision detection of biomarkers, physiological parameters, and environmental analytes.

## 3. Optical Sensing Mechanisms for Wearable Biosensors

Optical sensors have made significant progress in the field of wearable biosensors. Compared to traditional sensors, optical sensors offer unique advantages in real-time monitoring, non-invasive detection, high sensitivity, and multi-parameter monitoring [65]. The optical sensing mechanism primarily relies on phenomena such as light absorption, fluorescence, refraction, reflection, and resonance, utilizing optical transducers that interact with biomolecules to enable detection [66]. To ensure the long-term stability of wearable biosensors in dynamic human environments, the design of optical sensing mechanisms must balance both high sensitivity and the reliability of the sensors.

### 3.1. Principles of Flexible Optical Biosensors and Signal Conversion

Flexible optical biosensors operate by transducing physiological or biochemical variations into modulations of light intensity, wavelength, or phase, which are subsequently converted into measurable electrical signals. At the skin-device interface, physiological events such as pulsatile blood flow, tissue oxygenation, or analyte concentration induce changes in light absorption, scattering, or refractive index. For instance, photoplethysmography (PPG) relies on the Beer-Lambert law, where fluctuations in optical density caused by arterial blood volume variations generate the AC component of the PPG waveform, enabling continuous monitoring of heart rate and oxygen saturation [67,68]. In interferometric and resonance-based devices, strain or biochemical binding events lead to measurable phase shifts or resonance wavelength displacements, which can be correlated with analyte levels or biomechanical states [4].

The modulated optical signal is detected by a photodiode or phototransistor with wavelength-dependent responsivity, converting incident photons into photocurrent. This current is typically amplified through a transimpedance amplifier (TIA), filtered to suppress ambient light interference, and digitized by an analog-to-digital converter. Subsequent digital signal processing, such as band-pass filtering, motion artifact suppression, and feature extraction, translates the raw electrical output into physiologically relevant parameters, including heart rate, blood oxygen saturation, respiratory rate, and metabolite concentrations [11,35]. The use of flexible substrates, such as elastomers and hydrogels, ensures stable optical coupling under bending or stretching, thus maintaining high signal-to-noise ratios during dynamic motion. Collectively, this optical-to-electrical transduction pathway provides a reproducible and reliable framework for real-time, non-invasive health monitoring.

### 3.2. Surface Plasmon Resonance (SPR) and Localized Surface Plasmon Resonance (LSPR)

**Figure 3:**
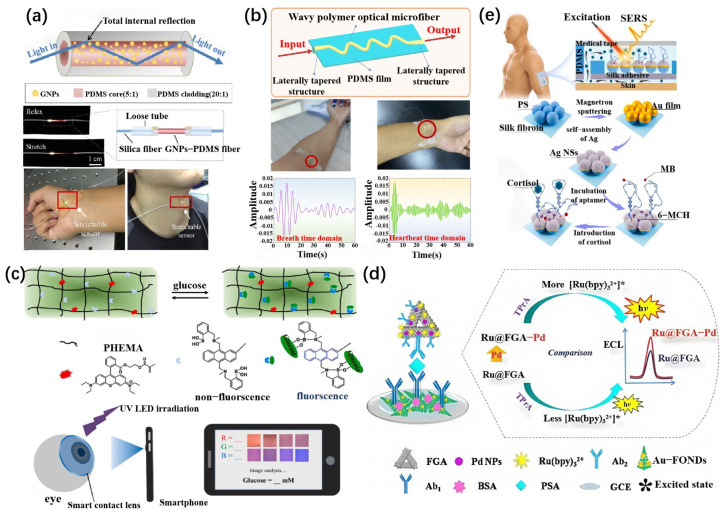
Representative applications of wearable optical biosensors based on different sensing mechanisms. (**a**) Schematic of a stretchable surface plasmon resonance (SPR) sensor constructed by embedding gold nanoparticles (GNPs) into a PDMS-based step-index optical fiber. The device enables stretch-induced modulation of plasmonic signals and has been applied for quantifying motor impairments in Parkinson’s disease patients [67]. (**b**) Wave-shaped optical microfiber sensor encapsulated in PDMS, fabricated via thermal drawing and used for non-invasive monitoring of respiration, heartbeat, and body movement [71]. (**c**) Fluorescent smart contact lens based on a PHEMA hydrogel matrix for real-time glucose monitoring. The device integrates with smartphones via image-based analysis under UV irradiation [23]. (**d**) Design and fabrication of an electrochemiluminescence (ECL)-based immunosensor array employing Ru@FGA-Pd nanocomposites, capable of enhancing ECL response for sensitive PSA detection [72]. (**e**) Schematic of a wearable sweat patch integrating a silk-based surface-enhanced Raman scattering (SERS) sensor with microfluidic channels. The platform enables real-time, label-free cortisol detection through aptamer-functionalized silver nanostructures [73].

The SPR is a technique that monitors refractive index changes by observing the oscillation of free electrons on a metal film surface and their interaction with biomolecules. It is widely used in high-sensitivity biosensors. SPR technology enables real-time detection of molecular interactions and offers significant sensitivity [69,70]. Recently, SPR sensors based on 2D materials such as MXenes and graphene have further enhanced both sensitivity and selectivity. For instance, Guo et al. designed an SPR-based strain sensor by embedding gold nanoparticles (GNPs) into a step-index elastomeric optical fiber (Figure 3a). The sensor uses a stretch-induced light attenuation mechanism to achieve a wide strain detection range from 0.09% to 100%. The sensor’s sensitivity reaches 9.54 dB/ε, and it can monitor finger bending angles and knee joint motion amplitude in real time, successfully applied to quantify motor impairment and tremors in Parkinson’s disease patients [67].

Similar to SPR, LSPR utilizes the localized electronic oscillation effect of metal nanoparticles to detect molecular interactions by measuring changes in light scattering or absorption. Compared to SPR, LSPR offers higher sensitivity and a broader operational wavelength range, making it particularly suitable for portable and wearable sensors [74]. Zhou et al. proposed a flexible sensor based on microwave plasmonic excitons (LSPR) to monitor blood glucose levels in real time. This sensor uses an ultra-thin flexible PET substrate and employs inkjet printing technology to fabricate metal corrugated ring structures, forming an LSPR sensor with extremely high sensitivity. The innovation of this technology lies in its ability to simulate the complex vascular environment of the human body, enabling real-time, non-invasive blood glucose monitoring with excellent wearability and long-term stability [75].

### 3.3. Optical Fiber Sensing Mechanism

Optical fiber sensors transmit signals through thin, flexible optical fibers made of high-quality glass or plastic. The surface of the optical fiber is typically modified with recognition elements such as antibodies or DNA, which can specifically bind to target analytes. When the analyte interacts with the recognition element, it induces a change in the evanescent wave, resulting in a variation in the light signal transmitted through the optical fiber. These changes can be detected and measured, allowing for the determination of the analyte concentration and achieving specific identification of the target analyte [76,77]. The primary sensing mechanisms in optical fiber sensors are index sensing and absorption sensing, each with distinct operating principles and applications.

Index sensing occurs when the refractive index of the surrounding medium changes, affecting the propagation characteristics of light within the fiber. These changes in the refractive index result in variations in the transmitted light’s intensity and/or wavelength. Index sensing is particularly sensitive to environmental changes such as temperature, pressure, and strain, making it highly suitable for applications like strain and temperature monitoring in structural health systems [63]. On the other hand, absorption sensing relies on the interaction between light and the target analyte, where the analyte absorbs light at specific wavelengths, leading to a measurable decrease in transmitted light intensity. This mechanism is particularly useful for detecting gases, liquids, and chemical pollutants, as the absorption characteristics are unique to the specific analyte [62]. Absorption sensing is less sensitive to environmental factors compared to index sensing but offers high specificity to particular compounds. While both mechanisms offer unique advantages, they also have distinct limitations. Index sensing is highly sensitive to physical changes in the environment but may be affected by non-analyte factors such as temperature or humidity. In contrast, absorption sensing provides excellent specificity for detecting targeted molecules but may require higher concentrations of analyte to achieve detectable changes in the signal. Both sensing mechanisms can be integrated into optical fiber sensor designs to enhance sensitivity, specificity, and versatility, depending on the particular application requirements [78].

Recent advancements in optical fiber sensors have made significant innovative advances in both design and application. For example, Cheng-Yu et al. employed fused deposition modeling (FDM) 3D printing technology to seamlessly integrate optical fiber Bragg gratings (FBG) into a flexible polylactic acid (PLA) ring substrate, creating a washable smart ring. This ring can precisely monitor the movement of the elbow and knee joints, achieving sensitivities of 0.0056 nm/° and 0.0276 nm/°, respectively, with maximum measurable angles of 90° and 100°. The 50% elongation at break of PLA ensures that the sensor can highly adapt to joint deformation [79]. In further research, Wang et al. proposed a flexible sensor based on wave-shaped optical fibers (Figure 3b). These fibers, with diameters ranging from 20 to 40 μm, are fabricated using a thermal stretching process and encapsulated in a PDMS wave-shaped structure, giving the sensor up to 100% tensile strain capability. After 1200 cycles of tensile testing, the wavelength and amplitude changes were only 0.43% and 0.56%, respectively, overcoming the brittleness and limited strain range of traditional silicon-based optical fibers. This sensor has been successfully applied to monitor cardiopulmonary function and joint movement, and it achieved 89% accuracy in medical keyword speech recognition when combined with artificial intelligence algorithms [71]. The advantages of optical fiber sensors lie not only in their high sensitivity and accuracy but also in their ability to simultaneously detect multiple analytes, enhancing both detection efficiency and data quantity. By integrating advanced materials (such as PLA and PDMS) and artificial intelligence algorithms, optical fiber sensors show great promise for flexible monitoring, medical diagnostics, and health surveillance applications.

### 3.4. Fluorescence Sensing Mechanism

Fluorescence sensors detect biomolecules by monitoring the interaction between target molecules and fluorescent probes, utilizing the optical properties of fluorescent markers. When the target molecule binds to the fluorescent probe, it typically induces changes in fluorescence intensity, lifetime, or wavelength, enabling quantitative analysis. Fluorescence sensors offer high sensitivity, real-time monitoring, and multi-parameter detection, making them particularly valuable in detecting low-concentration analytes [80,81]. As a result, they have become essential tools in non-invasive, high-precision biological diagnostics. For example, Deng et al. proposed a fluorescence-based smart contact lens designed for real-time blood glucose monitoring (Figure 3c). The lens uses a poly (hydroxyethyl methacrylate) (HEMA) hydrogel as a substrate material, covalently binding a glucose fluorescent probe with a reference fluorescent dye. The lens captures fluorescence signals via a smartphone, converting them into RGB signals for quantitative glucose concentration measurement, with a detection limit of 23 μM [23]. This technology overcomes the limitations of traditional blood glucose monitoring, offering a convenient, non-invasive, and highly sensitive solution for real-time glucose monitoring, with significant potential for clinical applications.

In another study, Yang et al. developed a fluorescence sensing system based on a PVA/CMC composite hydrogel. The system utilizes hydrogen-bonded crosslinking to construct a flexible three-dimensional network that demonstrates over 250% strain adaptability and pH-responsive dual-mode sensing capabilities. Within the pH range of 5–8, carbon quantum dots exhibit fluorescence blue shift, while the iodine-PVA composite undergoes visible light color changes. Using smartphone RGB analysis software, the system successfully monitors wound pH in situ. Additionally, the system integrates near-infrared temperature control and controllable iodine release functions, achieving an antibacterial efficiency of 92% and significantly accelerating cell migration. In mouse experiments, the system increased wound healing rates by 40%, providing an innovative solution for smart wound dressings [82]. Chen et al. combined fluorescence intensity and light transmission loss in a dual-parameter strategy, using the refractive index differences between methyl-phenyl-siloxane and PDMS to simultaneously monitor wrist joint motion angles and body temperature changes related to breathing patterns. This strategy effectively addressed the signal cross-sensitivity issue caused by motion interference, further improving the accuracy and reliability of the sensor [83].

### 3.5. Chemiluminescence and Electrochemiluminescence

Chemiluminescence sensors detect target molecules by utilizing light signals generated through chemical reactions. These light signals correlate with the concentration of the analyte, allowing for quantitative analysis. The advantages of these sensors include their high sensitivity and the lack of a need for an external light source. Electrochemiluminescence technology combines electrochemical reactions with light emission phenomena. It uses the interaction between luminescent compounds on the electrode surface and the target molecule to excite light signals. Both technologies offer extremely high sensitivity and precision, enabling real-time monitoring of dynamic biological molecule changes [84,85]. They are also capable of simultaneously detecting multiple analytes, making them particularly suitable for applications in personalized medicine and rapid diagnostics. Zhao et al. developed a dual-layer flexible “Test-to-Treat” patch, where the inner layer is composed of a hydrogel loaded with a pH indicator and an acid-responsive metal-organic framework (MOF). This patch enables rapid visual diagnosis through color changes (yellow indicating drug-sensitive bacteria and red indicating drug-resistant bacteria), significantly improving diagnostic speed and accuracy. The outer layer incorporates PDMS composite mechanoluminescent materials and photocatalysts (Pt@TiO_2_), providing the patch with flexible characteristics. By applying pressure or stretching, the patch triggers visible light emission, which subsequently drives photocatalytic reactions to generate reactive oxygen species (ROS). This design not only achieves a 99% bacterial inactivation rate but also offers a novel solution for rapid on-site diagnostics [86].

Yang et al. developed a novel Ru(bpy)_3_^2+^-based electrochemiluminescence (ECL) immunosensor utilizing palladium nanoparticle (Pd NP)-functionalized graphene-aerogel-supported Fe_3_O_4_ (FGA-Pd) for real-sample analysis of prostate-specific antigen (PSA) (Figure 3d). 3D nanostructured FGA-Pd, as a novel ECL carrier, was prepared by in situ reduction. Large amounts of Ru(bpy)_3_^2+^ could combine with FGA-Pd via electrostatic interaction to establish a brand-new ECL emitter (Ru@FGA-Pd) for improving ECL efficiency. The obtained Ru@FGA-Pd composite was utilized to label the secondary antibody, which generated strong ECL signals with tripropylamine (TPrA) as a core actant [72].

### 3.6. Surface-Enhanced Raman Spectroscopy (SERS)

The SERS utilizes the enhanced electromagnetic field of metal nanoparticles to significantly increase the intensity of Raman scattering signals, enabling the sensitive detection of molecules at very low concentrations. The integration of SERS technology with flexible substrates not only expands its range of applications but also improves the stability of the sensor in complex physiological environments [81,87]. For example, Mogera et al. proposed a flexible plasma paper-based microfluidic sweat sensor based on SERS, capable of continuous label-free detection of uric acid (UA) in sweat. The sensor achieved a detection limit of 1 μM, using stretchable double-sided tape, serpentine cellulose paper microfluidic channels, and an ultra-thin PDMS encapsulation layer. Even under 30% mechanical strain, the sensor maintained functional stability. During real-time monitoring of physical activities, the sensor was able to accurately detect UA concentrations in sweat, with a detection limit of 28 μM [88]. Hu et al. constructed a flexible SERS substrate through self-assembled double-layer nanosphere arrays (SFOS/Au/Ag NSs), combining the inherent adhesion properties of silk fibroin and a biomimetic structural design (Figure 3e). This significantly enhanced the mechanical stability of the sensor and its skin adhesion. The sensor was able to maintain stable signals under dynamic bending or pressure conditions, achieving a sensitivity of 33 pmol/L for cortisol detection in sweat [73]. Additionally, Lv et al. developed a biomimetic urchin-cavity SERS sensor, where the silver nanoparticle layer on a PDMS substrate interacts with copper oxide nanowires in a multidimensional electromagnetic field, maintaining 80% of the signal strength even when in contact with the skin. This sensor successfully detected urea at concentrations as low as 10^−10^ M and was able to measure diabetes-related acetone levels up to 30 mmol/L [89]. Flexible SERS-based sensors enable real-time, non-invasive disease detection.

In summary, optical sensing mechanisms have significantly advanced the capabilities of wearable biosensors, offering exceptional advantages in non-invasive, real-time, and multi-parameter monitoring. The choice of sensing mechanism is largely dictated by the specific requirements of the target application, including sensitivity, sample type, and the need for real-time detection. A comparative analysis of the key performance parameters—sensitivity, sample requirements, and application—of wearable optical biosensors is presented in Table 2. This table offers a comprehensive overview of the strengths and limitations of these sensing modalities, providing valuable insights for selecting the most suitable technology for diverse biosensing applications.

The subsequent chapter will explore the integration of flexible materials and optical sensing technologies, including sensor miniaturization, multi-functionalization, and the integration of smart features (e.g., wireless transmission, AI analytics), which are critical for translating lab-scale innovations into practical wearable devices.

## 4. Integration of Flexible Materials and Optical Sensing Technologies

The advent of flexible materials has opened new directions for the integration and application of optical biosensors. By combining flexible materials with advanced optical sensing technologies, researchers have developed more compact, convenient, and highly adaptable wearable sensors [96].

### 4.1. Miniaturization and Integration of Optical Sensors

**Figure 4:**
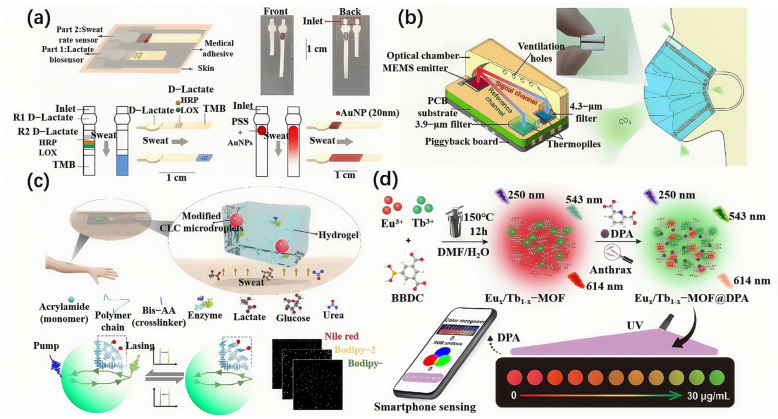
Integration of flexible materials with optical sensing technologies for wearable biosensors. (**a**) Schematic of a dual-function sensor for simultaneous detection of lactate concentration and sweat volume, demonstrating the integration of flexible materials with optical transducers for real-time biomarker monitoring [97]. (**b**) Diagram of an ultra-compact CO_2_ infrared gas sensor integrated into a wearable device, utilizing flexible substrates and optical sensing elements for continuous breath analysis [99]. (**c**) Conceptual schematic of a multifunctional flexible laser sensor based on modified cholesteric liquid crystal (CLC) droplets, highlighting the synergy between flexible materials and optical sensing mechanisms for sweat analysis [100]. (**d**) Illustration of the synthesis of Eu_x_/Tb_1−x_-MOF nanowires and their integration with smartphone-based ratiometric fluorescence sensing for the detection of DPA, showcasing the combination of flexible substrates with optical sensing for portable, real-time diagnostics [101].

The integration of optical sensors with flexible substrate materials such as PDMS, PET, and hydrogels not only enhances sensor portability but also enables lightweight designs through techniques like microfluidics and 3D printing, effectively reducing production costs. For example, Vaquer et al. designed a sensor for the simultaneous detection of lactate concentration and volume in sweat (Figure 4a), using flexible paper as the microfluidic channel and reaction carrier. The lightweight and flexible characteristics of the paper allow it to conform closely to the skin, ensuring stable collection during motion. This all-paper flexible sensor design offers multiple advantages, including low cost, customizability, and disposability [97]. Inamori et al. developed a wearable device based on colorimetric detection to monitor neonatal jaundice in real-time while also synchronously monitoring blood oxygen saturation and heart rate (HR). The device uses a PDMS lens to optimize light signal transmission, while the design is lightweight through 3D printing. Additionally, the flexible silicone base is designed to conform to the natural curvature of a neonate’s head, ensuring reliable continuous monitoring during phototherapy. This technology offers an innovative solution for precise neonatal jaundice management [98]. Feng et al. developed a miniaturized CO_2_ infrared sensor, which, although not directly utilizing flexible optical materials (Figure 4b), provides a technical paradigm for flexible module integration due to its 10 mm-sized packaging and 33 mW low-power consumption [99].

Davies et al. developed an ear canal-integrated photoplethysmogram sensor, whose core uses a viscoelastic foam earplug as a flexible substrate, embedding the chip and securing it with a 3D-printed shell. The flexible foam material adapts to the shape of the ear canal, ensuring stable contact and reducing wearing pressure, while also utilizing the ear canal’s natural electromagnetic shielding properties to suppress motion artifacts and low-temperature-induced vascular constriction [102]. Liu et al. developed a wearable dual-wavelength diffuse speckle contrast blood oxygenation monitor based on flexible materials, designed for simultaneous monitoring of blood flow and oxygenation changes in deep tissues. This device uses a 3D-printed polylactic acid (PLA) probe, integrating a micro-laser diode and CMOS camera, and replaces traditional rigid optical fibers with flexible cables, significantly improving wearability and movement freedom. The design also includes a thermistor to monitor skin temperature in real-time, ensuring safety during long-term use. The device’s measurement accuracy was validated through in-vivo experiments and forearm arterial occlusion tests, showing high agreement with commercial devices [103]. These innovations provide ultra-flexible, passive solutions for wearable blood oxygen saturation monitoring.

### 4.2. Development of Multifunctional Sensors

In recent years, the multi-functionalization of flexible optical biosensors has become a key area of research. By integrating different sensing modules, such as optical sensing, electrochemical sensing, and temperature sensing modules, into a single substrate, researchers have achieved multi-parameter monitoring capabilities. For example, Nie et al. developed a flexible polyacrylamide (PAM) hydrogel-based thin-film laser sensor that integrates both optical and electrochemical sensing modules to achieve real-time multi-parameter monitoring of skin metabolites (Figure 4c). This sensor has detection sensitivities of 0.15 nm/mM for lactate, 0.002 nm/μM for glucose, and 0.14 nm/mM for urea, with detection limits covering the physiological concentration range of human sweat [100]. Liu et al. developed a hydrogel-optical-electronic multimodal sensor that utilizes transparent conductive hydrogel as both a strain-resistive and light propagation channel. This design solves the issue of signal coupling in traditional multimodal sensors and enables more efficient integration of different sensing modes. The sensor is encapsulated with a highly elastomeric VHB™ film combined with black silicone to successfully shield external light interference. Additionally, by incorporating the BiLSTM algorithm, it decouples the stretching and bidirectional bending composite deformation signals, ensuring accurate monitoring of wrist flexion, extension, and adduction movements, with an error rate of less than 7.2% [104]. This multifunctional sensor can simultaneously detect various physiological parameters, providing comprehensive health data and supporting personalized medicine and disease early warning systems.

### 4.3. Integration of Smart Features and Wireless Transmission Technologies

With the continuous advancement of wireless communication technologies and artificial intelligence, flexible optical biosensors are progressing toward smarter and remote monitoring capabilities. By connecting sensors with smartphones or other mobile devices, users can view physiological data in real-time and manage their health, thereby improving personal health monitoring and disease prevention efficiency. For instance, Wang et al. developed an optical biosensor for detecting Bacillus anthracis infections (Figure 4d). This sensor enables real-time monitoring via a smartphone’s RGB analysis software, and the sensor also integrates machine learning and data analytics algorithms, which automatically identify abnormal signals and provide health alerts [101].

Deng et al. developed a smartphone-based fluorescence-sensing contact lens that changes color from pink to blue as glucose concentration increases. When connected to a smartphone, the fluorescence image is captured and converted into RGB signals for quantitative measurement, enabling rapid blood glucose detection within 3–5 s. Compared to existing electrochemical contact lenses, this technology does not require enzyme assistance, uses transparent flexible materials, and offers portable detection through a smartphone, addressing issues associated with rigid materials and complex equipment reliance in traditional electronic sensors [23]. Günther et al. integrated Ag/AgO_X_-TiO_2_ hybrid materials into a flexible wristband, which, through LSPR colorimetric responses, combined with a smartphone, enables the visualization of formaldehyde detection at 5 ppb levels [105].

In addition, the technical achievements of Dong et al. provide new technical support for the development and application of wearable optical biosensors. They designed and validated a stretchable self-powered triboelectric nanogenerator sensor array (TENG-SA) based on polyacrylamide (PAAM)/NaCl conductive hydrogel and silicone rubber. The PAAM/NaCl hydrogel has a maximum stretchability of 307%, the highest gauge factor (GF) of 18.4 for the strain sensor, and a light transmittance of 73% at a wavelength of 550 nm, enabling motion detection of human body parts such as the wrist, elbow, knee, and neck without an external power supply. By integrating a long short-term memory (LSTM) neural network (with a training accuracy of 90%), an intelligent sensing system was constructed. This system uses neck motion signals transmitted via Bluetooth to control the movement of a two-wheeled robot, successfully achieving human–robot interaction (HRI) [106]. In the future, integrating flexible sensors with microprocessors can expand the application of HRI in the medical and industrial fields. This smart integration enhances the potential application and market competitiveness of flexible optical biosensors in personalized health monitoring and disease prevention.

To summarize, integrating flexible materials with optical sensing has enabled breakthroughs in sensor miniaturization, multi-functionalization, and smart connectivity. However, despite these advances, critical hurdles remain in material stability, long-term wearability, and cost-effectiveness that hinder widespread adoption. The following chapter will address challenges in the development of wearable optical biosensors, systematically analyzing issues such as material degradation under dynamic environments, skin irritation risks, signal interference, and scalable manufacturing, and providing insights into potential solutions.

## 5. Challenges in the Development of Wearable Optical Biosensors

### 5.1. Material Stability and Performance Under Environmental Conditions

The stability of wearable optical biosensors is a key factor influencing their long-term performance. Since these sensors must operate continuously in the human body and various complex environmental conditions, the material stability and resistance to environmental interference directly determine the sensor’s lifespan and reliability.

Many materials used in wearable optical biosensors, such as graphene, transition metal dichalcogenides (TMDCs), and hydrogels, although possessing excellent electrical and optical properties, suffer from poor chemical stability and are vulnerable to environmental humidity and temperature. For example, graphene exhibits a more than 15% attenuation in SPR signals after being exposed to humidity above 60% for 24 h, significantly decreasing its sensitivity [107]. Additionally, MXene materials, under dynamic stretching conditions, can experience interfacial delamination due to differences in thermal expansion coefficients, thereby compromising the stability of the sensor signal [108,109]. Conductive hydrogels are also affected by environmental factors: in dry environments, the loss of water content can cause the hydrogel’s structure to become brittle and shrink, altering the optical signal’s transmission path and the characteristics of the detection interface [110]. Similarly, flexible materials like PDMS experience a reduction in transparency under high-temperature conditions. When stretched by 50%, the transparency drops by approximately 8%, which affects the wavelength shift precision of LSPR-based sensors [111].

In addition to temperature and humidity, environmental pollution, particularly chemical pollutants such as ammonia and chemical warfare agents, can also impact the performance of optical biosensors. Research has shown that PDMS-based nanofiber sensors enhance gas diffusion efficiency through porous structures and effectively block liquid interference using hydrophobicity, allowing stable operation in extreme environments. However, sensors still face sensitivity degradation in complex air pollutant environments, which highlights the need for further optimization of the environmental adaptability of sensor materials [110]. To address these issues, structural design strategies such as athermal resonator geometries and slot-waveguide layouts have been reported to mitigate thermally induced index shifts, reducing temperature sensitivity while preserving compact form factor. In parallel, Similar strategies using polymer-inorganic hybrid encapsulation have been shown to suppress moisture permeation and maintain optical transparency under cyclic bending [112,113]. Recent studies have demonstrated the feasibility of ultrathin organic-inorganic integrated devices that couple organic electrochemical transistors with near-infrared µLEDs on conformal substrates, enabling wireless optical biomarker monitoring with high stability and biocompatibility [114]. These solutions highlight how tailored optical structures combined with encapsulation layers can improve environmental resilience, though at the expense of increased fabrication complexity. Future research should focus on material surface modification, composite design, and systematic assessments of environmental adaptability to improve the stability of wearable optical biosensors under extreme conditions. At the same time, the integration of on-chip light sources (e.g., micro-light-emitting diodes, µLEDs) and the implementation of advanced packaging strategies represent essential aspects to ensure long-term device reliability.

### 5.2. Biocompatibility and Long-Term Wearability

Biocompatibility and long-term wearability represent another major challenge for the successful application of wearable optical biosensors. These sensors need to be in continuous contact with the skin, particularly in dynamic movement scenarios, where comfort and biocompatibility are crucial. If the sensor material causes allergic reactions or discomfort upon contact with the skin, it may result in a poor user experience, potentially compromising the reliability of health monitoring.

Many materials used in sensors, such as MXenes, metallic nanoparticles (e.g., silver nanoparticles), and hydrogels, may release harmful ions or trigger skin allergic reactions. For instance, silver nanoparticles may release Ag^+^ ions when exposed to sweat, causing cytotoxic reactions on the skin [115]. Additionally, natural polymers like silk fibroin and cellulose, although exhibiting excellent biocompatibility, have poor mechanical strength, making them unsuitable for the durability requirements in complex motion scenarios [116]. Therefore, material selection must carefully evaluate the long-term safety of these materials, particularly in extended skin contact conditions.

Compared to traditional sensors, wearable optical biosensors are typically designed for extended use, making comfort and convenience critical considerations in their design. Flexible materials such as PDMS, polyimide (PI), and polyethylene terephthalate (PET) are widely used in wearable devices due to their good mechanical properties and ability to adapt to dynamic changes in skin surfaces [117]. However, during long-term wear, factors like material deformation, surface friction, and the penetration of skin sweat may cause discomfort and decrease wearability stability. To overcome these limitations, structural designs incorporating breathable mesh allow conformal skin interfaces while enhancing flexibility and comfort [118]. At the packaging level, engineered composite fabrics with multilayered structures offer waterproofing and high moisture permeability, while antimicrobial coatings provide both user comfort and robust bactericidal performance, thereby reducing irritation during prolonged wear [119]. These strategies significantly improve skin compatibility and long-term wear stability, although they may slightly increase manufacturing complexity and device thickness. Taken together, future research should focus on addressing challenges related to friction between flexible materials and the skin, breathability, and sweat permeability, as well as minimizing skin irritation and enhancing moisture management, while ensuring that these materials maintain high sensitivity, durability, and stability over extended periods of use in various environmental conditions.

### 5.3. Signal Interference and Sensitivity Optimization

Signal interference and sensitivity optimization are core issues affecting the performance of wearable optical biosensors. Since wearable optical biosensors need to detect physiological signals in real-time amidst complex environmental and human activities, the stability and accuracy of the signals are influenced by various factors, such as motion artifacts, electromagnetic interference, and structural changes in the sensor.

In wearable environments, sensors are often affected by motion artifacts and electromagnetic interference. Traditional optical sensors typically use rigid materials, making them less adaptable to the dynamic movement of the human body, leading to signal fluctuations and interference [120]. For instance, optical biosensors based on PDMS substrates experience changes in transparency and signal stability when bent [121]. The introduction of flexible materials has alleviated some of the rigid constraints of traditional sensors, but challenges remain in ensuring signal stability in dynamic movement environments.

Sensitivity is an important indicator for optical biosensors, particularly for detecting disease markers and early diagnosis, where the ability of the sensor to effectively capture weak biological signals determines its success [122]. Currently, the sensitivity of flexible optical biosensors is still limited by the optical response properties of materials and the structural design of the sensors. For example, LSPR-based sensors, while having high sensitivity, exhibit significant attenuation in signal strength under stretching and bending conditions [123]. Recent strategies have demonstrated that incorporating sensor arrays coupled with artificial intelligence-assisted algorithms can effectively suppress noise and enhance recognition accuracy, thereby improving sensitivity and real-time stability in wearable applications [124]. Additionally, the integration of gallium-based liquid metal nanodroplets into MXene nanosheets has been shown to increase interlayer spacing, improve mechanical robustness, and maintain stable signal transmission under stress, providing a promising approach to mitigate deformation-induced signal degradation [125].To optimize sensitivity, future research can focus on several aspects: developing flexible materials with stronger optical response capabilities, such as improving the surface chemistry of fibers, metallic nanoparticles, or MXenes; and adopting more precise manufacturing techniques, such as nanoimprinting and laser etching, to enhance the resolution and signal-capturing ability of sensors.

### 5.4. Cost and Manufacturing Considerations

While significant progress has been made in enhancing the performance, stability, and biocompatibility of wearable optical biosensors, their practical translation ultimately depends on cost-effectiveness and scalable manufacturing. The fabrication cost is primarily influenced by factors such as substrate materials, functional inks, patterning and post-processing steps, encapsulation, and end-of-line testing, with device yield further amplifying these costs. For example, noble-metal-based inks, such as silver, are expensive, while lower-cost alternatives, such as copper or carbon nanomaterials, offer potential for cost reduction. However, these alternatives require reliable oxidation control and performance stabilization to ensure their viability [126].

Roll-to-roll and other printing-based techniques present viable pathways to large-scale, flexible device fabrication. Phung et al. demonstrated that roll-to-roll screen printing can reduce both manufacturing time and costs for IoT devices [127]. The printing methods offer lower material costs and reduced waste, making them attractive for flexible electronics [128]. Moreover, emerging fabrication methods, including printed electronics, soft transfer, and 3D printing, provide enhanced design freedom and system integration for wearable devices, facilitating scalable production while meeting form-factor requirements [129].

However, advanced encapsulation and multi-step manufacturing approaches that improve environmental robustness often introduce higher processing complexity and cost. Therefore, future work must integrate cost-control strategies such as material substitution, thickness and geometry optimization, and low-energy post-processing, alongside design-for-manufacturing approaches that simplify assembly and minimize calibration requirements. Industrial-scale deployment of wearable optical biosensors for real-world healthcare applications.

In summary, the development of wearable optical biosensors is constrained by four core challenges: material stability under complex environments, biocompatibility for long-term wear, signal interference in dynamic scenarios, and cost barriers for mass production. Addressing these issues requires not only material and structural innovations but also a forward-looking perspective on future research directions. The final chapter will present conclusions and interdisciplinary insights, synthesizing the progress of flexible materials and optical sensing technologies, and proposing future pathways to accelerate the translation of wearable optical biosensors into clinical and consumer healthcare applications.

## 6. Conclusions

Flexible optical biosensors have shown immense promise in personalized healthcare, health monitoring, and disease prevention. With the continuous advancement of flexible materials, optical sensing technologies, and intelligent algorithms in recent years, the performance and functionality of these sensors have been significantly enhanced. Flexible materials, due to their excellent biocompatibility, mechanical adaptability, and lightweight nature, have become fundamental components in wearable sensors. In particular, these sensors offer significant advantages in non-invasive detection and real-time monitoring, establishing them as essential tools for future health management and personalized medicine.

In this review, we have systematically summarized the recent progress in flexible optical biosensors, with particular attention to substrate and functional materials, sensing mechanisms, and representative applications. The discussion highlights that substrate materials such as polymers, hydrogels, textiles, and thin inorganic films play a decisive role in determining the flexibility, thermal stability, and biocompatibility of wearable sensors [28]. Functional materials, including metallic nanostructures, carbon-based nanomaterials, and MXenes, offer superior optical and electrical properties that significantly enhance detection sensitivity [51]. Moreover, diverse optical transduction mechanisms, such as fluorescence, surface plasmon resonance, surface-enhanced Raman scattering, and interferometry, enable the realization of sensors with high sensitivity and specificity under dynamic conditions.

However, despite substantial advancements, several challenges persist. First, the stability of sensor materials under complex environmental conditions remains a critical concern. Factors such as temperature fluctuations, humidity, and environmental pollution may impact the long-term reliability of these sensors. Therefore, further efforts are required to optimize the environmental adaptability of materials, enhancing their durability and stability under extreme conditions. Second, biocompatibility and long-term wearability remain challenges for wearable optical biosensors, especially when in prolonged contact with the skin. The potential for materials to cause allergic reactions or discomfort can negatively impact user experience and the reliability of health monitoring. Addressing these concerns requires the development of softer, more comfortable, and biocompatible materials to enhance user comfort. Moreover, signal interference and sensitivity optimization remain critical to the overall performance of these sensors. In dynamic environments, motion artifacts, electromagnetic interference, and structural changes in the sensors can affect signal stability. As such, future designs must optimize the signal processing capabilities of flexible optical biosensors to ensure their stability during movement. Additionally, improving the sensitivity of these sensors—particularly in detecting low-concentration biological signals—will be crucial for early disease diagnosis and precision medicine.

Research from related fields provides important insights for future development. For example, studies on mechanically responsive metamaterials have demonstrated that through precise structural design and multi-stimulus response mechanisms (such as thermal, electrical, optical, and magnetic stimuli), materials can achieve high sensitivity and tunability in complex dynamic environments. This concept offers new inspiration for wearable optical biosensors to maintain stable performance under continuous motion and environmental fluctuations [130]. Meanwhile, advances in photomultiplication-type organic photodetectors highlight the unique advantages of organic semiconductors in achieving optical signal self-amplification, reducing noise interference, and enhancing weak signal detection capability. These developments align closely with the practical needs of optical biosensors in detecting low-concentration biomarkers [131]. Future research on flexible optical biosensors can be inspired by these two directions: on the one hand, adopting the design strategies of metamaterials that leverage structure-driven functionalities, combining geometric designs with flexible materials to achieve efficient responses to complex stimuli; on the other hand, introducing the signal amplification principles of organic photo-detecting materials to improve sensitivity and reliability under low-light and low-concentration detection conditions. The interdisciplinary integration of these new approaches is expected to drive the emergence of next-generation flexible optical biosensing platforms and accelerate their practical applications in health monitoring, early diagnosis, and personalized medicine.

At the same time, future research should focus on the multi-functionalization and integration of materials, with an emphasis on enhancing the intelligence and wireless transmission capabilities of sensors. By integrating artificial intelligence, machine learning, and big data analytics into flexible optical biosensors, the accuracy of data and the real-time processing of information can be further improved. This will enable the broader application of personalized health management and disease prevention. In conclusion, while flexible optical biosensors continue to evolve, the combination of innovative materials and cutting-edge technologies holds great promise for the field. By overcoming current challenges and advancing technological developments, flexible optical biosensors will play an increasingly significant role in personalized healthcare, intelligent health monitoring, and early disease diagnosis in the future.

## Figures and Tables

**Table 1:** Representative flexible materials for wearable optical biosensors and their main characteristics.

	**Material** **Category**	**Flexibility**	**Thermal Stability**	**Electrical Conductivity**	**Biocompatibility**	**Fabrication Process Specialty**	**Ref.**
**Substrate Materials**	PDMS	Excellent stretchable	Low (<150 °C)	Insulating (needs composites)	Excellent	Replica molding/soft lithography; microfluidics integration	[28,31,32]
		Advantages: Low processing cost; Simple molding process; Optically transparent. Disadvantages: Low thermal stability; Hydrophobic surface.					
	PI	Moderate	High (>300 °C)	Insulating	Good, slightly brittle	Spin coating; photolithography; thin-film metallization	[29,64]
		Advantages: Good electrical insulation; Radiation resistance; High chemical stability. Disadvantages: Yellowish color, poor transparency; High brittleness; Prone to cracking under large deformation.					
	PET	Good, bendable, non-stretchable	Moderate (~150 °C)	Insulating	Moderate, needs surface treatment	Roll-to-roll printing; Inkjet/screen/gravure printing	[30,59]
		Advantages: High transparency; Low cost; Smooth; Mature roll-to-roll processing. Disadvantages: Poor biodegradability; Environmentally constrained; Polar surface, requires modification.					
	Hydrogels	Highly elastic, soft	Poor (temp sensitive)	Ionic conduction	Excellent, degradable	Photo/covalent/ionic crosslinking; 3D printing; dopant/biopolymer integration	[53,54,55]
		Advantages: Tissue-like softness; High water content; Conformal adhesion; Ionic conduction; Transparency. Disadvantages: Pure hydrogel has low ionic conductivity; Dehydration/freeze sensitivity.					
	Textiles	High, conformable	Variable (cotton < 200 °C; aramid > 400 °C)	Needs conductive yarns/coatings or ionic conduction	Good, breathable	Screen printing; embroidery/knitting of conductive yarns	[36,59,60]
		Advantages: Breathable; Conformable; Scalable; Hierarchical porosity aids sweat sampling. Disadvantages: Surface roughness/variability; Patterning challenges; Wash durability issues.					
**Functional Materials**	Noble Metal Nanoparticles	Achieved via Compliant substrates/nano-meshes, particles themselves rigid	Good for Au; Ag less stable in sulfur/halide environments	Metallic (high)	Au generally good, Ag dose/size dependent-needs passivation	Nanoimprint/e-beam; transfer printing to elastomers/textiles	[39,40]
		Advantages: High chemical stability; Surface plasmon effect. Disadvantages: High cost; Nanoparticles prone to agglomeration; High process requirements for bonding with flexible substrates.					
	Carbon-based Nanomaterials	Excellent	High in inert; oxidize > ~400–500 °C	High	Generally good after functionalization, dose/aggregation dependent	CVD growth; screen/inkjet/spray printing; laser reduction of GO	[41,42,43]
		Advantages: Large specific surface area; Lower cost. Disadvantages: Prone to stacking and agglomeration; Weak interfacial bonding with polymer substrates; High-cost.					
	MXene	Excellent as few-layer films/papers and coatings	Moderate; oxidation risk in high temperature	High	Promising but formulation/termination dependent	Selective etch of MAX; intercalation and surface functionalization	[48,49,50]
		Advantages: Rich surface functional groups; Excellent electrochemical performance; High specific capacitance. Disadvantages: Ambient oxidation; Requires encapsulation for long-term stability.					
	Thin-film of Inorganic Non-metallic Material	Good when t ≲ 100–500 nm and placed near neutral plane, small bending radius achievable	High for many oxides/chalcogenides (often >300 °C)	Semiconducting or insulating (tunable by doping/phase)	Generally good for oxides, composition-dependent	Lift-off/transfer to elastomers; CVD/ALD/sol-gel	[62,63]
		Advantages: Maintains superior optical/electronic properties; Flexible at nanoscale; Low optical. Disadvantages: Limited strain; Complex/expensive processing; Passivation often required.					

**Table 2:** Comparison of the key performance characteristics of wearable optical biosensors.

**Biosensor**	**Sample Requirements**	**Detection Range (M)**	**Sensitivity**	**Cycling Stability**	**Application**	**Ref.**
**SPR**	Low sample volume; Label-free; Real-time detection	10^−12^–10^−6^	High Sensitivity, 10^3^–10^5^ RIU^−1^	High (>50 cycles with regeneration)	Biomarker detection (proteins, DNA, small molecules); Clinical diagnostics	[67,75,90]
**Optical Fiber** **Sensors**	Low sample volume; Label-free; Real-time detection	10^−9^–10^−5^	Moderate to high Varies by Principle, ~10^−8^ M LOD	High (>50 cycles, depending on coating)	In vivo monitoring; Wearable biosensing; Environmental detection	[71,79,91]
**Fluorescence** **Sensors**	Low sample volume; Require fluorescent labeling; Real-time possible	10^−9^–10^−3^	High Sensitivity, ~10^−7^ M LOD	Moderate (signal drift after 5–10 cycles)	Enzyme assays; Immunoassays; Intracellular imaging	[23,82,83]
**Chemiluminescence/** **Electrochemiluminescence Sensors**	Low sample volume; often label-free; Real-time detection not available	10^−12^–10^−9^	~10^−12^ M LOD (ECL highly sensitive)	High (>100 cycles with stable electrode)	Clinical diagnostics (cardiac markers, cancer biomarkers, nucleic acids)	[86,92,93,94]
**SERS**	Low sample volume; Label-free; Real-time detection	10^−12^–10^−9^	Sensitivity single-molecule level (10^6^–10^8^ enhancement)	Moderate (substrate degradation after ~20 cycles)	Ultrasensitive detection of pathogens, DNA, proteins; Point-of-care diagnostics	[73,88,89,95]

## Data Availability

Not applicable.

## Abbreviations

The following abbreviations are used in this manuscript:

PDMS	Polydimethylsiloxane
PI	Polyimide
PET	Polyethylene terephthalate
CRP	C-reactive protein
VSC	Volatile sulfur compounds
0D/1D/2D	Zero/One/Two-dimensional
SPR	Surface plasmon resonance
LSPR	Localized Surface Plasmon Resonance
CNTs	Carbon nanotubes
MWCNTs	Multi-walled carbon nanotubes
CPs	Polyimide aerogel-based paper
ECL	Electrochemiluminescence
CVD	Chemical vapor deposition
IFE	Internal filter effect
PEG	Polyethylene Glycol
PAM	Polyacrylamide,
PVA	Polyvinyl alcohol
pHEMA	Poly(hydroxyethyl methacrylate)
pSB	Poly(sulfobetaine)
PVDF	Polyvinylidene fluoride
PAHS	Polycyclic aromatic hydrocarbons
UV	Ultraviolet
GGFF	Graphene-coated glass fiber fabric
POF	Polymer optical fibers
GNPs	Gold nanoparticles
FDM	Fused deposition modeling
FBG	Fiber bragg gratings
PLA	Polylactic acid
HEMA	Hydroxyethyl methacrylate
CMC	Carboxymethyl cellulose
MOF	Metal-organic framework
ROS	Reactive oxygen species
PSA	Prostate specific antigen
SERS	Surface-enhanced raman spectroscopy
HR	Heart rate
CMOS	Complementary metal-oxide-semiconductor
TMDCs	Transition metal dichalcogenides
